# A novel combined approach to placement of a double lumen endobronchial tube using a video laryngoscope and fiberoptic bronchoscope: a retrospective chart review

**DOI:** 10.1186/s12871-024-02525-6

**Published:** 2024-04-12

**Authors:** Luiz Maracaja, Alexandra Coffield, L. Daniela Smith, J. David Bradshaw, Amit K. Saha, Christopher S. McLauglin, T. Wesley Templeton

**Affiliations:** 1 Department of Anesthesiology, Wake Forest University School of Medicine, Medical Center Boulevard, Winston-Salem, Charlotte, NC 27157-1009 USA; 2 Department of Anesthesiology, Duke University School of Medicine, Durham, NC USA

**Keywords:** One-lung ventilation, Thoracic anesthesia, Double lumen endobronchial tube, Airway management

## Abstract

**Background:**

The objective of this study was to evaluate a modern combined video laryngoscopy and flexible fiberoptic bronchoscope approach to placement of a double lumen endobronchial tube and further characterize potential strengths and weaknesses of this approach.

**Methods:**

Retrospective chart review was conducted at our single institution, academic medical center, tertiary-care hospital. Patients aged 18 years of age or older were evaluated who underwent thoracic surgery and one-lung ventilation with placement of a double lumen endobronchial tube using a novel combined video laryngoscopy and flexible fiberoptic bronchoscope approach. No interventions were performed.

**Results:**

Demographics and induction and intubation documentation were reviewed for 21 patients who underwent thoracic surgery and one-lung ventilation with placement of a double lumen endobronchial tube using a novel combined video laryngoscopy and flexible fiberoptic bronchoscope approach. First pass success using the combined approach was 86% (18/21). The five patients with an anticipated difficult airway had successful double lumen endobronchial tube placement on the first attempt. There were no instances of desaturation during double lumen endobronchial tube placement. No airway complications related to double lumen endobronchial tube placement were recorded.

**Conclusion:**

Use of a combined approach employing video laryngoscopy and a flexible fiberoptic bronchoscope may represent a reliable alternative approach to placement of double lumen endobronchial tubes.

**Supplementary Information:**

The online version contains supplementary material available at 10.1186/s12871-024-02525-6.

## Introduction

Lung isolation and one-lung ventilation (OLV) are used to provide surgical exposure for a variety of commonly performed thoracic procedures. At present, double lumen endobronchial tubes remain the gold standard and the most commonly used device for OLV in adults [1, 2]. The placement and positioning of double lumen endobronchial tubes can be significantly more challenging than placement of standard endotracheal tubes, particularly in settings where there is a more anterior glottis, and/or the mouth opening is even mildly limited. The larger external diameter of double lumen endobronchial tubes as well as their increased stiffness compared to standard endotracheal tubes can make them more difficult to maneuver in the airway. Also, the orifice of the tracheal lumen of the double lumen endobronchial tube in some cases will behave like an open bevel during passage through the glottis, impinging on the arytenoids and the posterior larynx. This can impede advancement during placement and potentially lead to airway mucosal abrasions. Combined, these issues may contribute to reduced first chance success rates for placement of double lumen endobronchial tubes and potentially lead to increased risks of adverse events including in rare cases airway rupture [3].

Consequently, we have developed a novel approach to placement of a double lumen endobronchial tube, which combines both video laryngoscopy and a flexible fiberoptic bronchoscope. This combined approach has previously been well-established in the setting of managing the adult difficult airway [4]. Typically, this is executed using a video laryngoscope to expose and view the glottis while at the same time using a flexible fiberoptic bronchoscope to enter the glottis while being viewed with the video laryngoscope. In this approach, the flexible fiberoptic bronchoscope becomes almost a steerable stylet over which the endotracheal tube can be railroaded once the flexible fiberoptic bronchoscope is in the trachea.

Therefore, the primary aim of this study was to evaluate this technique further in a larger swath of patients via a retrospective chart review to characterize potential strengths and weaknesses of this approach to double lumen, endobronchial tube placement including complications related to airway management.

## Methods

This retrospective review was approved by the Advocate – Wake Forest University School of Medicine Institutional Review Board (IRB #93,096), and a waiver of consent was granted given the retrospective nature of the study. This manuscript was developed using the applicable Enhancing the Quality and Transparence of Health Research Guideline (EQUATOR) checklist.

The local electronic health record (EHR) was queried for elective, thoracic cases in patients aged 18 years or older. Inclusion criteria were patients aged at least 18 years undergoing thoracic surgery and OLV with placement of a double lumen endobronchial tube using a combined video laryngoscopy and flexible fiberoptic bronchoscope approach. Patients who were under the age of 18 and for whom an alternative approach to double lumen, endobronchial tube placement was used were excluded. Demographics including age, weight and body mass index (BMI), sex, American Society of Anesthesiologists (ASA) Physical Status classification, type and side of procedure were recorded. Induction and intubation documentation, as well as airway and miscellaneous notes were reviewed. Induction agent and dosing, type of neuromuscular blocker and dosing, size of double lumen endobronchial tube, and number of attempts at double lumen, endobronchial tube placement were recorded. The number of additional episodes of mask ventilation and desaturation events during double lumen, endobronchial tube placement, if present, was recorded. An episode of desaturation was defined as any oxygen saturation (SpO_2_) value < 90% for 1 min or more in the EHR during airway management defined as the period from induction to successful intubation.

All patients presented initially as outpatients or were pre-admitted for scheduled surgery. Patients were brought to the operating room and following adequate pre-oxygenation, they underwent an intravenous induction with propofol and rocuronium. Patients were then intubated using a combined approach. All intubations were performed by resident physicians supervised by one of two board-certified attending anesthesiologists. Importantly, this approach requires two clinicians to execute. Double lumen endobronchial tube sizes were chosen per patient at the discretion of the attending anesthesiologists, based on patient demographics, cricoid cartilage diameter, and respective bronchial diameter.

**Fig. 1:**
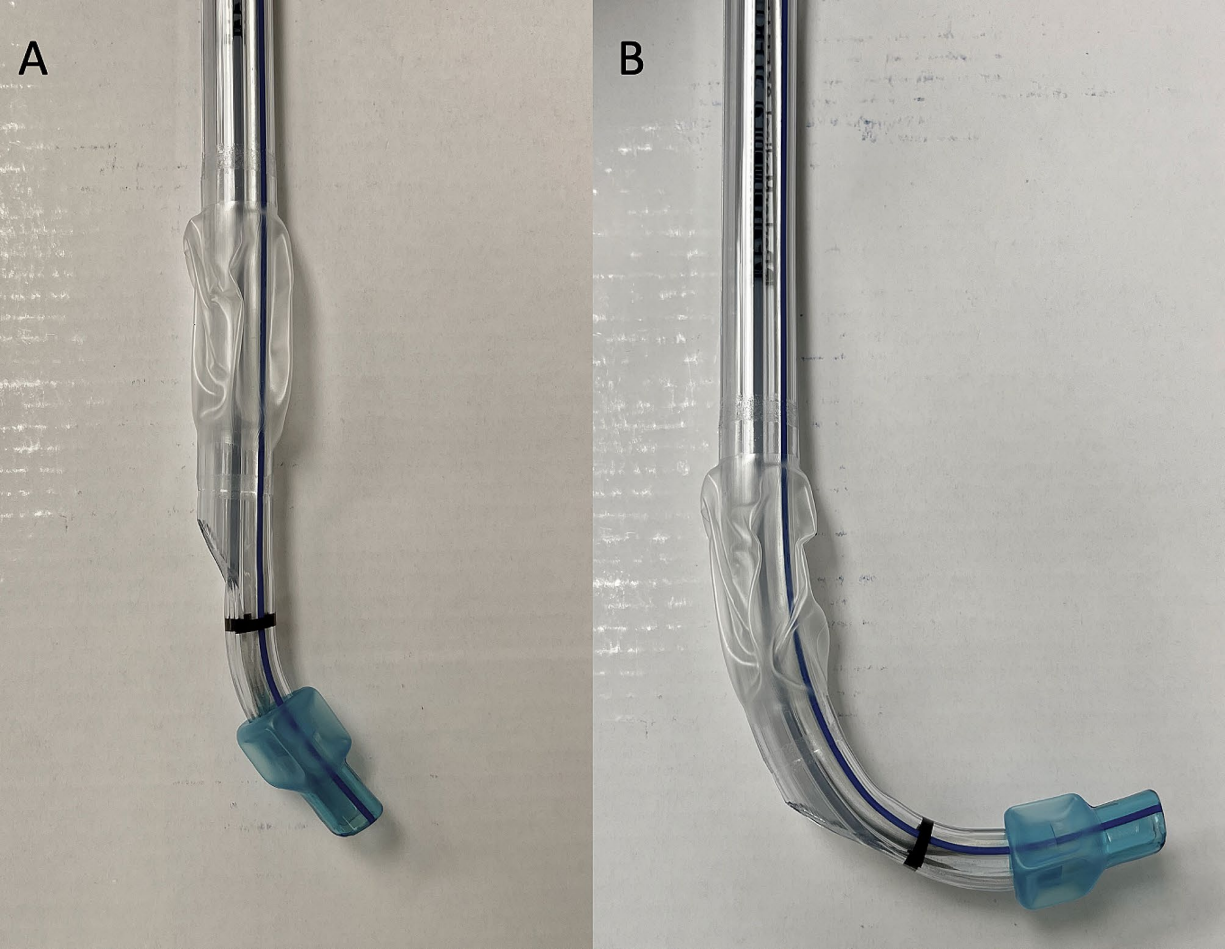
(**A**) Left sided double lumen endobronchial tube. (**B**) Left-sided, double lumen endobronchial tube with approximate 90 degree bend at the distal end

**Fig. 2:**
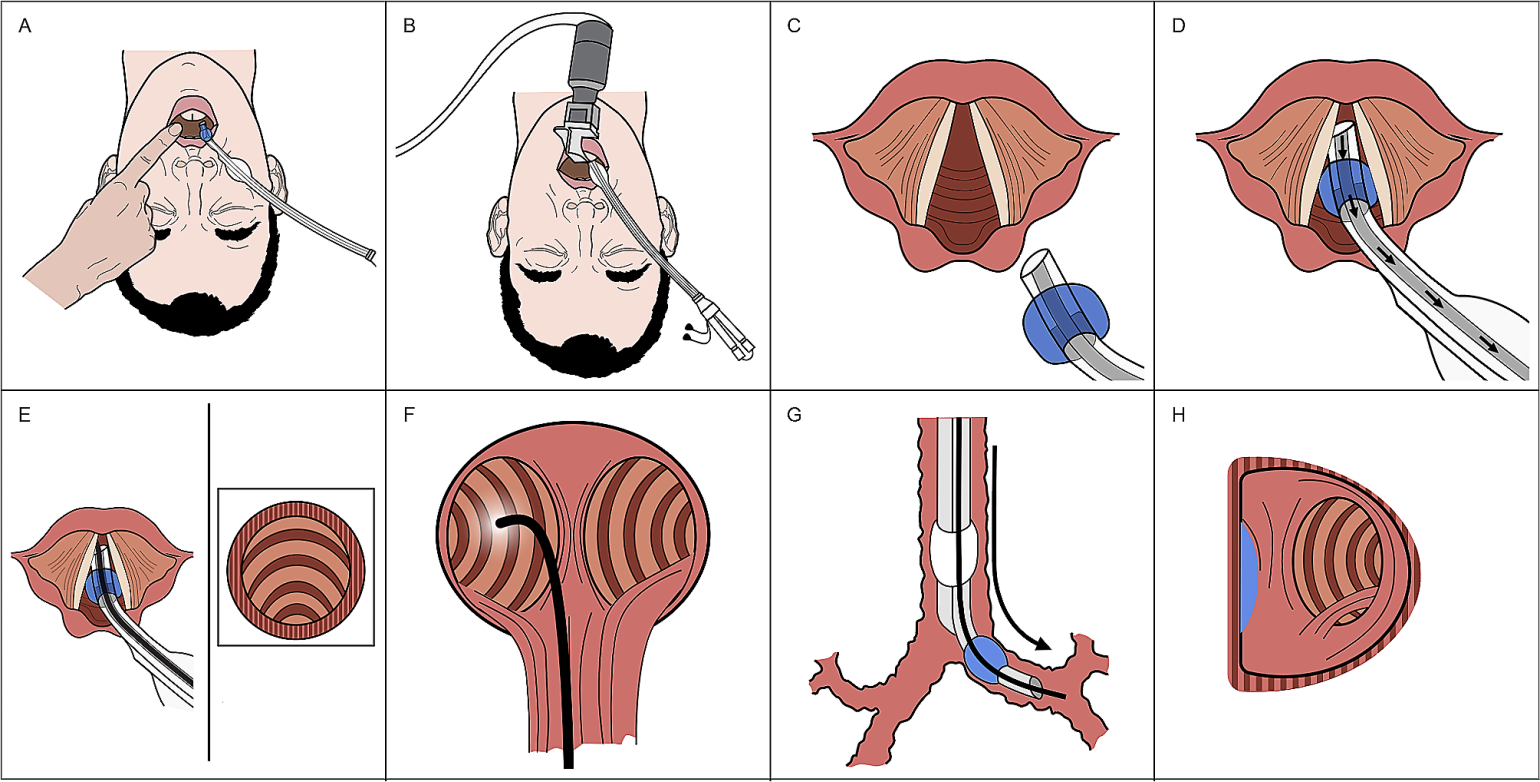
(**A**) Double lumen endobronchial tube is inserted into the mouth in the midline and then shifted to the right side of the mouth. (**B**) A video laryngoscope is inserted. (**C**) The bronchial lumen and bronchial cuff are advanced into the glottis. (**D**) Once below the vocal cords the stylet is removed from the double lumen endobronchial tube. (**E**) A fiberoptic scope is introduced into the bronchial lumen and the trachea is imaged. (**F**) The fiberoptic bronchoscope is advanced into the left mainstem bronchus. (**G**) The double lumen endobronchial tube is advanced over the fiberoptic scope and the bronchial lumen and cuff are advanced into the left mainstem bronchus. (**H**) The fiberoptic scope is inserted into the tracheal lumen and final adjustment to the depth of the double lumen endobronchial tube is performed so that a rim of the bronchial cuff can be seen at the carina

The first step in this combined approach was to place the double lumen endobronchial tube into the patient’s oropharynx in the midline with the included stylet in place. The stylet and distal end of the double lumen, endobronchial tube should be bent approximately 90 degrees prior to insertion (Fig. 1**)**. The distal end of the double lumen endobronchial tube was then gently moved to the right side of the oropharynx and held in place by an assistant; leaving the center of the oropharyngeal cavity open and accessible for placement of the video laryngoscopy blade. During initial attempts at refining this combined approach, we found that starting with the double lumen, endobronchial tube in the midline, prior to placing the video laryngoscopic blade in the midline, allowed for a reduction in manipulation necessary to align the distal end of the double lumen, endobronchial tube with the glottis, once it was shifted to the right of the oropharynx. Next, the video laryngoscope blade was placed into the oropharyngeal cavity and a view of the glottis was obtained. If possible, the bronchial cuff of the double lumen endobronchial tube was then passed through the vocal cords under indirect visualization via the video laryngoscope. The stylet of the double lumen endobronchial tube was then removed, and the fiberoptic bronchoscope was advanced through the bronchial lumen into the trachea, past the carina and into the desired mainstem bronchus. If it is not possible to get the endobronchial cuff into the glottis because of malalignment, the flexible fiberoptic bronchoscope can be inserted into the endobronchial lumen at this point and can be driven into the glottis under indirect view with the video laryngoscope. The double lumen, endobronchial tube was then railroaded over the flexible fiberoptic bronchoscope into the appropriate mainstem bronchus. At this point, the video laryngoscope was removed from the mouth and the flexible fiberoptic bronchoscope was inserted into the tracheal lumen to assess and finalize bronchial cuff depth. An overview of this technique can be seen in Supplemental Video 1. Following placement, typical clinical assessments of correct tracheal placement including observation of chest wall expansion, auscultation, and the presence of end-tidal CO_2_ were performed. The double lumen, endobronchial tube was then secured in place and the patient was positioned in the lateral decubitus position. Once the patient was positioned completely in the lateral position, the flexible fiberoptic bronchoscope was re-introduced in both lumens to confirm proper positioning and depth of the double lumen, endobronchial tube. Once confirmed, the bronchial cuff was inflated, the bronchial lumen was clamped, and the lung was allowed to collapse passively by absorption atelectasis. A schematic summary of this combined approach to double lumen, endobronchial tube placement is shown in Fig. 2.

Descriptive statistics were performed on the baseline characteristics of all patients. Data are presented as means and standard deviations.

## Results

A query of the local EHR yielded 21 patients who underwent thoracic procedures with OLV using this combined approach to double lumen, endobronchial tube placement. Patient demographics are summarized in Table 1. Most patients underwent right-sided procedures (13 of 21). The first chance success rate in this cohort was 86% (18/21). Three of the 21 cases had more than one attempt at double lumen, endobronchial tube placement (one with two attempts and two with three attempts), with one of those cases requiring a single additional period of mask ventilation between attempts. There was no documentation of desaturation events in the EHR during double lumen, endobronchial tube placement for any of the cases.

**Table 1:** Demographic information and airway exam summary for patients undergoing one-lung ventilation with a double lumen endobronchial tube using a video laryngoscope and a flexible fiberoptic bronchoscope

	*N* = 21
Age, yrs ± SD	62.8 ± 13.6
Weight, kg ± SD	90.1 ± 22.6
Body mass index ± SD	30.3 ± 6.9
Male/female	13/8
ASA physical status 2, 3, 4	1, 15, 5
Anticipated difficulty airway	5
Mallampati I, II, III	3, 10, 7
Neck ROM full/limited	17/3
TMD (cm) 1, 2, 3	**1, 18, 1**

*Abbreviations* ASA, American Society of Anesthesiologists Physical Status Classification; ROM, range of motion; TMD, thyromental distance

In the five patients noted to have an anticipated difficult airway, we were able to successfully place a double lumen, endobronchial tube using this combined approach on the first attempt. A breakdown of the technique by individual, as well as details of their procedure and double lumen, endobronchial tube placement, is presented in Table 2. No airway complications related to endobronchial tube placement were noted in any patients.

**Table 2:** Individual case data for patients undergoing one-lung ventilation with a double lumen endobronchial tube using a video laryngoscope and a flexible fiberoptic bronchoscope

Patient ID	Age (yr)	Weight (kg)	Operative Side (L/R)	Anticipated Difficult Airway (Y/N)	Size of DLT (Fr)	# of Attempts at Placement	# of Additional Episodes of BVM Required
1	64	103.4	L	N	39	1	0
2	78	59	R	N	39	1	0
3	66	111.6	R	N	39	1	0
4	68	119.7	R	N	39	3	1
5	55	98	R	Y	39	1	0
6	69	108.9	L	Y	39	1	0
7	54	109.8	L	N	35	1	0
8	77	62.6	L	N	35	1	0
9	35	91.6	R	N	39	1	0
10	54	103	R	N	39	1	0
11	63	59.4	R	Y	35	1	0
12	54	57.1	R	N	37	1	0
13	25	108.9	R	N	35	1	0
14	83	76.2	L	N	37	1	0
15	68	99.3	R	N	35	1	0
16	68	84.4	L	Y	35	1	0
17	66	110.2	R	N	41	2	0
18	72	122.5	R	Y	39	1	0
19	65	53.5	L	N	35	1	0
20	60	83	R	N	35	1	0
21	74	68.9	L	N	37	3	0

*Abbreviations* L, left; R, right; Y, yes; N, no; DLT, double lumen endobronchial tube; Fr, French catheter scale; BVM, bag-valve mask ventilation

## Discussion

The primary finding of this retrospective chart review was that the novel approach of using a combination of video laryngoscopy and a flexible fiberoptic bronchoscope likely represents a reliable alternative approach to placement of double lumen endobronchial tubes. Additionally, double lumen, endobronchial tube placement could, in most cases with this approach, be achieved on the first attempt.

Strengths of this approach stem from its deliberate focus on eliminating or minimizing common complications and difficulties with double lumen, endobronchial tube placement. The video laryngoscope blade is thicker than that of a standard laryngoscope in many cases, and when combined with the larger diameter of the double lumen, endobronchial tube, the oral cavity and pharynx can become crowded with little room to maneuver. One randomized controlled trial comparing the GlideScope to the Macintosh laryngoscope found that anesthesiologists with significant experience in thoracic anesthesia and use of the GlideScope for endotracheal tube insertion found double lumen endobronchial tube placement with the GlideScope to be more difficult as compared with the Macintosh laryngoscope [5]. In this study, the most difficult steps associated with use of the GlideScope for intubation with a double lumen, endobronchial tube were reported to be insertion of the device and tube into the mouth and passing the tube through the cords. We have found that our approach, with placement of the double lumen, endobronchial tube into the oropharynx prior to placement of the blade, helps to offset or even eliminate the difficulty brought about by the video laryngoscope blade while still allowing for the benefit of its use for visualization of the glottis. This novel approach may offer an advantage over other previously described techniques to combat the difficulty associated with double lumen, endobronchial tube insertion using video laryngoscopy, which have been focused mainly on stylet manipulation and tube angle modification prior to tube insertion [6–9]. Despite report of increased difficulty associated with video laryngoscopy for double lumen, endobronchial tube insertion, video laryngoscopy in these cases may offer shorter intubation times, a higher intubation success rate, decreased cardiovascular response, and frequently a better glottic view as compared to direct laryngoscopy [8, 10, 11].

Notably, there were no reported airway complications, pharyngeal, or tracheal injuries with the use of this combined technique, with a minority of patients requiring more than one attempt. There were no documented instances of post-operative throat discomfort or hoarseness in the post operative electronic health record although these would not typically be documented unless they were fairly significant. As a result mild or moderate sore throats/hoarseness may not have been documented. Generally, the advancement of the double lumen, endobronchial tube through the airway tended to be smoother with the use of the combined approach, a result of the bronchoscope keeping the tube in the center of the airway, lessening the chance of the tip leading to tracheal or bronchial mucosal abrasions. Interestingly, although fiberoptic bronchoscopy is generally considered the gold standard for confirmation of proper positioning of double lumen, endobronchial tubes, there is a relative paucity of literature describing the use of a bronchoscope to aid with double lumen endobronchial tube placement during intubation [12–15]. Hou-Chuan et al. describe an approach that uses both video laryngoscopy and fiberoptic bronchoscopy, with introduction of the fiberoptic scope into the tracheal lumen of the double lumen tube rather than the bronchial lumen [12]. This approach permits direct visualization of the advancement of the bronchial lumen into the desired mainstem bronchus. Our approach, while similar in its combined use of video laryngoscopy and fiberoptic bronchoscopy, may offer an advantage in its use of the fiberoptic bronchoscope as a stylette that can be driven directly into the desired mainstem bronchus. Taken altogether, our combined approach may represent an avenue to decrease the incidence of tracheobronchial injury associated with double lumen, endobronchial tube placement in some cases. However, due to the small sample size and lack of a defined control or comparison group, no definite conclusion can be drawn as to the relative incidence of these complications without further study. The combined technique allows continuous video laryngoscopy of the glottic entrance while the DLT is advanced into the trachea. The continuous visualization enables the rotation of the DLT to avoid the arytenoid cartilage (Supplemental Video 1).

The main advantage of the combined technique is the ability to guide the tip of the DLT during insertion into the tracheal bronchial tree. Using the traditional technique the tip of the DLT will be directed to the wall of the trachea. However, using the combined technique the bronchoscope in most cases maintains the tip of the DLT in the center of the airway leading to potentially less airway trauma. This can be visualized in Supplemental Video 2. While this combined approach may offer advantages over more traditional approaches to double lumen, endobronchial tube placement in specific instances, it may not be suitable for use in certain patient populations or clinical scenarios. For example, secondary to reliance on video laryngoscopic and/or fiberoptic bronchoscopic view, this technique would not be suitable in situations involving active bleeding in the airway such as in the case of double lumen, endobronchial tube placement for bronchial artery embolization for hemoptysis [10]. Although we were successful in the five patients with a known difficult airway, limited mouth opening, limited neck range of motion, and short thyromental distance could potentially affect use of this approach. Interestingly, this novel technique was specifically beneficial in one instance in our dataset, where unsuccessful placement using direct laryngoscopy was ultimately followed by successful placement using the combined approach (Patient ID 21). In this case, the Cormack Lehane grade view was determined to be grade I with utilization of the video laryngoscope, after initial documentation of a grade III view using a Macintosh-3 blade. While there could have been a multiplicity of potential issues, the ultimate success of this approach in this case demonstrates the technique’s ability to help visualize specific aspects of double lumen, endobronchial tube placement, while at the same time using a “smart stylet” in the form of a flexible fiberoptic bronchoscope, which may lead to increased rates of success when more conventional approaches fail.

Limitations of the study include the small sample size and its retrospective nature. Additionally, there was no defined control group reviewed for comparison. The intubation time was also not systematically recorded, secondary to the retrospective nature of this study, and as such, one cannot comment on whether this technique would affect total intubation time, which may be of clinical significance in patients undergoing thoracoscopic surgery who frequently may have diminished pulmonary reserve. Of note, however, there were no reported desaturation events during intubation in the study population. Also, rates of success may vary depending on the type of video laryngoscope used as there has been a proliferation of various manufacturers and designs which may ultimately impact the efficacy of this approach. Finally, we cannot necessarily infer the rate of success of this approach in patients with a known difficult airway or in cases where specific airway indices may impact intubation success in different ways, for example in the setting of limited mouth opening versus limited neck mobility. However, this approach may prove helpful in some of these circumstances. Though this approach was found to aid in the successful placement of a DLT in five patients who were predicted to have potentially difficult airways, it should be noted that none of these patients had airway indices which led to consideration of awake intubation. If, on pre-operative evaluation of a patient, the attending anesthesiologist had deemed the patient’s airway indices to be predictive of both difficult ventilation and difficult intubation, then an awake intubation would’ve been chosen as the preferred strategy in that patient. In that scenario, DLT placement with our technique would likely not have been feasible. As such, this does represent a potential limitation of our technique.

In conclusion, this novel combined approach to placement of a double lumen, endobronchial tube using a video laryngoscope and a flexible fiberoptic bronchoscope was largely successful with no airway related complications and a high first attempt rate of success. Further prospective research in a larger cohort of patients evaluating this combined technique in comparison to other commonly employed strategies for double lumen, endobronchial tube placement may garner further insights.

### Electronic supplementary material

Below is the link to the electronic supplementary material.

Supplementary Material 1

Supplementary Material 2

## Data Availability

The data that support the findings of this study are not openly available due to institutional privacy guidelines but may be available from the corresponding author upon reasonable written request identifying the requestor, and the purpose and proposed use of the shared data.

## Abbreviations

ASA: American Society of Anesthesiologists
BMI: body mass index
EHR: electronic health record
OLV: one-lung ventilation
SpO_2_: oxygen saturation
